# Improving phylogenetic resolution of the Lamiales using the complete plastome sequences of six *Penstemon* species

**DOI:** 10.1371/journal.pone.0261143

**Published:** 2021-12-15

**Authors:** Jason M. Stettler, Mikel R. Stevens, Lindsey M. Meservey, W. Wesley Crump, Jed D. Grow, Sydney J. Porter, L. Stephen Love, Peter J. Maughan, Eric N. Jellen

**Affiliations:** 1 Department of Plant and Wildlife Sciences, Brigham Young University, Provo, Utah, United States of America; 2 Department of Biology, Stanford University, Stanford, California, United States of America; 3 Department of Horticulture, Irrigated Agriculture Research and Extension Center, Washington State University, Prosser, Washington, United States of America; 4 Department of Plant Sciences, North Dakota State University, Fargo, North Dakota, United States of America; 5 Aberdeen Research and Extension Center, University of Idaho, Aberdeen, ID, United States of America; G. B. Pant Institute of Himalayan Environment & Development, INDIA

## Abstract

The North American endemic genus *Penstemon* (Mitchell) has a recent geologic origin of ca. 3.6 million years ago (MYA) during the Pliocene/Pleistocene transition and has undergone a rapid adaptive evolutionary radiation with ca. 285 species of perennial forbs and sub-shrubs. *Penstemon* is divided into six subgenera occupying all North American habitats including the Arctic tundra, Central American tropical forests, alpine meadows, arid deserts, and temperate grasslands. Due to the rapid rate of diversification and speciation, previous phylogenetic studies using individual and concatenated chloroplast sequences have failed to resolve many polytomic clades. We investigated the efficacy of utilizing the plastid genomes (plastomes) of 29 species in the Lamiales order, including five newly sequenced *Penstemon* plastomes, for analyzing phylogenetic relationships and resolving problematic clades. We compared whole-plastome based phylogenies to phylogenies based on individual gene sequences (*matK*, *ndhF*, *psaA*, *psbA*, *rbcL*, *rpoC2*, and *rps2*) and concatenated sequences. We also We found that our whole-plastome based phylogeny had higher nodal support than all other phylogenies, which suggests that it provides greater accuracy in describing the hierarchal relationships among taxa as compared to other methods. We found that the genus *Penstemon* forms a monophyletic clade sister to, but separate from, the Old World taxa of the *Plantaginaceae* family included in our study. Our whole-plastome based phylogeny also supports the rearrangement of the *Scrophulariaceae* family and improves resolution of major clades and genera of the Lamiales.

## Introduction

The genus of *Penstemon* (Mitchell) is a large group of ca. 285 species of flowering plants endemic to North America [1–3]. A recently released report by Wolfe, Blischak (3) hypothesize the origin for *Penstemon* around the Pliocene/Pleistocene transition ca. 3.6 million years ago (MYA) [3]. This genus has extraordinary genetic and phenotypic diversity as evidenced by the array of ecosystems it inhabits and number of published taxa [4]. Geographic distribution ranges from the Yukon River Basin of Alaska and Canada to the Yucatan Peninsula of Mexico and Guatemala. The rapid and recent diversification and speciation in *Penstemon* is associated with dramatic differences in diploid genome size ranging from 462 megabase pairs (Mbp) in *P*. *dissectus* to 922 Mbp in *P*. *nitidus* [5]. The rate of speciation, span in genome sizes, and relatively low mutation rate in plastome coding regions creates a unique challenge for systematists attempting to fully resolve phylogenetic relationships in *Penstemon*.

Recent phylogenetic analyses of many *Penstemon* taxa using a small number of diagnostic plastid genetic regions indicated that some of these subgenera may be poly- or paraphyletic [1, 2, 6]. As a result, Freeman (2019) proposed that the subgenera be reduced to two: *Dasanthera*, reconstructed by combining *Dasanthera* and *Cryptostemon*; and *Penstemon*, reconstructed by combining *Saccanthera*, *Dissecti*, *Habroanthus*, and *Penstemon* [7]. Regardless, most *Penstemon* taxonomists continue to use the historic subgenera classification as work continues to resolve problematic clades (i.e. gene tree discordance, polytomy, polyphyly, paraphyly, etc.) within and among subgenera and sections [8–10].

Phylogenetic investigations of Scrophulariaceae, the family to which *Penstemon* was previously described, using the *rbcL* and *ndhF* plastid genes revealed that the family was polyphyletic which led to extensive taxonomic revision of this family and a reorganization of the Lamiales order including placing the genus *Penstemon* into the Plantaginaceae family [11–13]. These revisions have been verified through multiple independent studies using other combinations of plastid genes [12–15]. Basal nodes of the Lamiales lineages have consistently had a high degree of resolution and nodal support in these phylogenetic studies. However, lineages inclusive of more recent diversification typically have poor resolution and low nodal support (polyphyly, paraphyly, and polytomies)–such as observed in *Penstemon* [2] and *Plantago* [16]–and are typified by low levels of variation in individual gene sequences [17].

Due to their maternal inheritance, magnoliid chloroplast genomes are conserved within each Order and Family [18] and are characterized by low rates of mutation/nucleotide substitution and recombination, plastid gene sequences are useful for studying the evolutionary history of land plants [19–21]. However, most phylogenetic analyses utilize few plastid genes to classify taxa, with *matK*, *ndhF*, *rbcL*, and *rps2* genes among the more common sequences used [11, 22, 23]. Selective pressure typically varies between genes, causing varying substitution rates and introduces data bias in observed in phylogenetic inference of individual gene sequences [19, 24]. Phylogenetic resolution can be improved, however, by using multiple concatenated genes [25–27], non-coding regions including introns [28–30], and partial genomic regions (i.e. long single-copy, short single-copy, inverted repeat, etc.) [31].

Historically, phylogenetic studies used few sequences and/or taxa due to the limiting cost of sequencing, and the computational demand of sequence assembly, alignment, and analyses. In recent years, the cost and ease of DNA sequencing, along with advances in computational power and algorithms for genome assembly and phylogenetic analyses have improved to the point that, as of January 2021, there have been 4,650 Viridiplantae plastomes published on the National Center for Biotechnology Information (NCBI) website (www.ncbi.nlm.nih.gov). These plastomes represent 4,616 unique taxa from 1,795 genera. In 2020 alone there were 1,165 new plastomes were published. Yet this valuable resource is underutilized in evolution evolutionary and phylogenetic studies, as the majority of plastome assembly announcements publish phylogenies based on concatenated sequences.

The process of examining multiple chloroplast gene sequences (individually or concatenated) to infer maximum likelihood (ML) phylogenetic relationships treats each character as independent. However, this violates the assumption of independence inherent in ML analyses as each locus is dependent and linked on single heritable plastid chromosome that does not undergo recombination [32, 33]. Basing phylogenetic analyses on whole organellar sequences rather than individual gene sequences does not violate the assumption of independence. This also improves phylogenetic resolution and confidence can be improved using whole-plastome sequences because the coding, noncoding, single-copy (long and short), and inverted repeat regions each accumulate point mutations at different rates within a plastome [34]. This then creates a phylogeny that represents the whole evolutionary history of the taxa based on the plastid genome.

Here we report the assembled and annotated plastomes of *P*. *cyaneus*, *P*. *dissectus*, *P*. *palmeri*, *P*. *personatus*, *and P*. *rostriflorus*, representing the *Habroanthus*, *Dissecti*, *Penstemon*, *Cryptostemon*, and *Saccanthera* subgenera, respectively. We include the previously published plastome of *P*. *fruticosus* [35] in our analyses to represent the basal subgenus, *Dasanthera*, thus including representatives from all recognized *Penstemon* subgenera. We had three primary objectives to guide our studies of *Penstemon* plastomes. First, document the complete plastome sequences for species representing each *Penstemon* subgenus. Second, evaluate these species for evolutionarily significant similarities and differences in plastome structure, using microsatellite simple sequence repeats (SSRs), repetitive sequences, nucleotide variants, expansion/contraction of the inverted repeats, plastome synteny, and the phylogenetic positions of the subgenera within *Penstemon*. Lastly, evaluate the efficacy of using whole-plastome sequences to resolve problematic clades within the Lamiales (i.e. gene tree discordance, polytomy, and low nodal support) when compared to phylogenies based on single-gene *matK*, *ndhF*, *psaA*, *psbA*, *rbcL*, *rpoC2*, and *rps2* sequences and a concatenated sequence composed of the above listed genes [11–13].

## Materials and methods

### DNA extraction, sequencing, and plastome assembly and annotation

**Fig 1:**
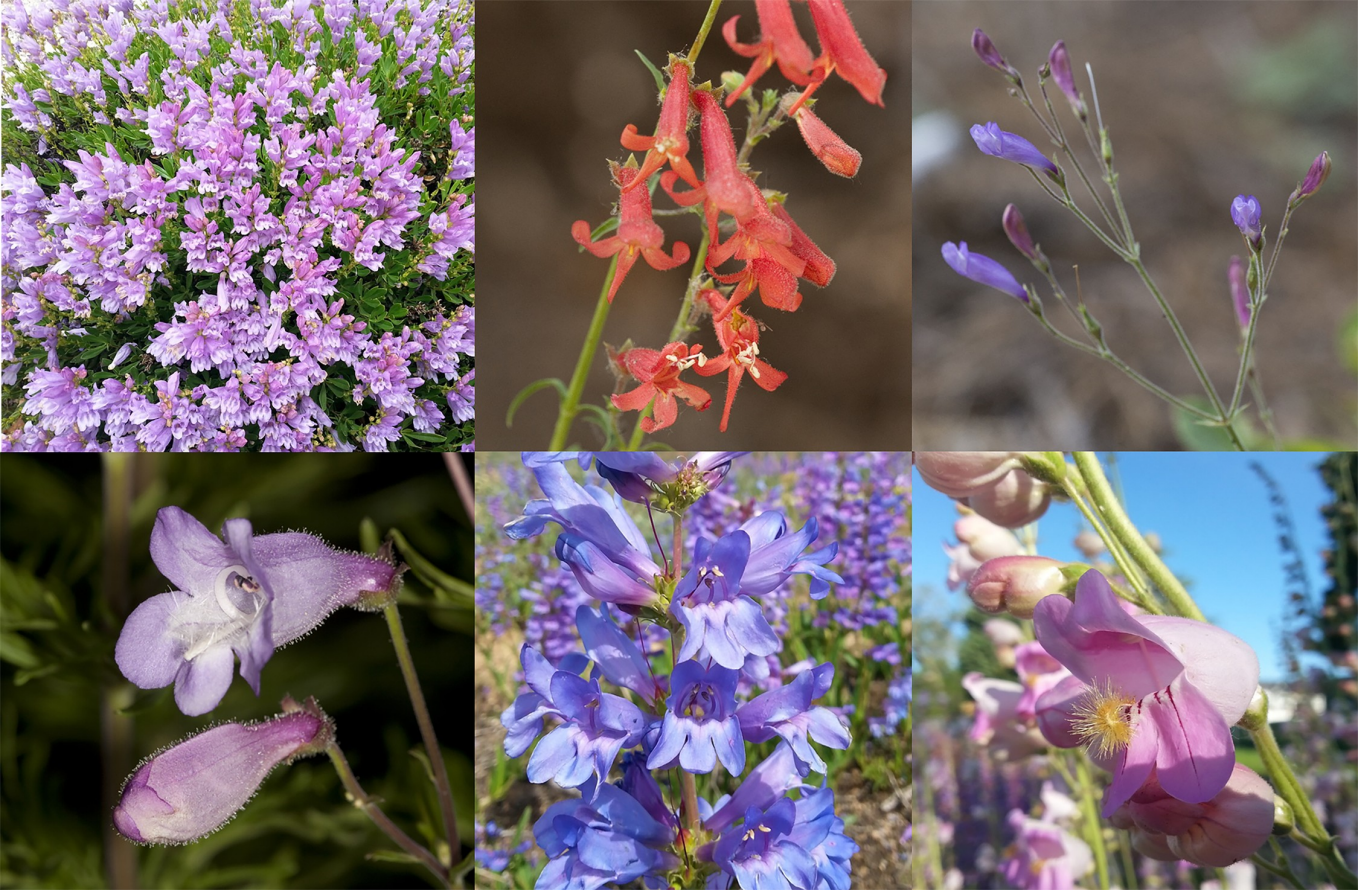
Photos of each species and respective subgenus included in this study. Clockwise from the upper left: *Penstemon fruticosus* (*Dasanthera*), *P*. *rostriflorus* (*Saccanthera*), *P*. *personatus* (*Cryptostemon*), *P*. *palmeri* (*Penstemon*), *P*. *cyaneus* (*Habroanthus*), *and P*. *dissectus* (*Dissecti*). Photo credits: Mikel R. Stevens and Jason M. Stettler.

We extracted DNA from leaves of greenhouse grown *P*. *cyaneus*, *P*. *dissectus*, *P*. *fruticosus*, *P*. *palmeri*, *P*. *personatus*, *and P*. *rostriflorus* (Fig 1) using a modified CTAB purification method [36]. We diluted the DNA to a minimum concentration of 5 ng and whole genome sequences were generated using the pair-end (2 x 250 bp) Illumina HiSeq platform (Illumina Inc., San Diego, CA) at the Brigham Young University DNA Sequencing Center (Provo, Utah, USA).

To isolate the plastome sequences from the unpaired genomic reads with NOVOPlasty (https://github.com/ndierckx/NOVOPlasty) [37] we used four combinations of seed inputs and reference plastomes: 1) *P*. *fruticosus* as the seed without a reference 2) *P*. *fruticosus* for both the seed and reference, 3) *Erythranthe lutea* as the seed without a reference, and 4) *E*. *lutea* as the seed and *P*. *fruticosus* as the reference. Then, we assembled the isolated sequences from all NOVOPlasty runs using the Geneious v11.0.3 De Novo Assembly tool [38] with the *P*. *fruticosus* plastome reference [35] to create a circularized plastome sequence. Next, we evaluated each assembly for coverage and identified sequence gaps (*P*. *cyaneus* and *P*. *palmeri*) and filled these gaps with additional NOVOPlasty runs using the portion of the *P*. *fruticosus* plastome sequence that spanned each gap as a seed reference.

We annotated the assembled plastomes using the GeSeq webserver (https://chlorobox.mpimp-golm.mpg.de/geseq.html) [39] with the default parameters, and NCBI reference sequences from *Olea europaea*, *Scrophularia buergeriana*, *S*. *takesimensis*, *Veronica nakaiana*, *V*. *persica*, and *Veronicastrum sibiricum*, and corrected any gene annotations that were missing start/stop codons or introns manually. Once we completed the annotations, we submitted all sequences and the annotations to NCBI.

### Simple sequence repeats and repeat structure

To evaluate the assembled plastomes for SSRs and repeat region similarity among *Penstemon* subgenera we used the MISA microsatellite predicting webserver (http://misaweb.ipk-gatersleben.de/) [40]. Our identified SSRs were based on homopolymer and copolymer lengths of five for di-, four for tri-, and three for tetra-, penta- and hexa-nucleotide repeats using the default parameters. Using the REPuter webserver (https://bibiserv.cebitec.uni-bielefeld.de/reputer) we identified repeat regions in the forward and reverse direction as well as complement and palindrome sequences [41]. We used a 20 base pair minimum repeat size without a minimum distance between repeat sites.

### Single nucleotide polymorphisms and codon preference

For the alignment of all sequences to the *P*. *fruticosus* reference we utilized MAFFT webserver (https://mafft.cbrc.jp/alignment/server/) [42] and identified the variable sites, SNPs and indels, with Geneious v11.0.3 [38]. To measure the relative synonymous codon usage (RSCU), we used the “Codon Usage Bias” function of DnaSP v6 [43], defined as the ratio between the frequency of use to expected frequency for each codon [44] within aligned protein coding DNA sequence (CDS).

### Synteny blocks

To examine structural variants at the inverted repeat (IR) region junction sites, we employed IRScope webserver (https://irscope.shinyapps.io/irapp/) [45] and visually evaluated the structural variants within the *Penstemon* genus and within a subset of taxa from the Lamiales order (Table 1). To do this, we downloaded the GenBank files for each taxon (Table 1) from NCBI for the IRScope input. The IRScope webserver processes ten plastomes at a time, so we made several submissions of plastome groups to compare the IR junctions of all taxa using the *Solanum lycopersicum* GenBank file as an outgroup reference for uniform alignment of each submission.

**Table 1:** Lamiales order and outgroup taxa included in our phylogenetic analyses.

Taxon	Family	Order	Plastome Length	CG %	NCBI Reference	Publication Year
*Solanum lycopersicum*	*Solanaceae*	Solanales	155,461	37.86	NC_007898.3	2014
*Haberlea rhodopensis*	*Gesneriaceae*	Lamiales	153,099	37.80	NC_031852.1	2016
*Streptocarpus teitensis*	*Gesneriaceae*	Lamiales	153,207	37.60	NC_037184.1	2018
*Salvia japonica*	*Lamiaceae*	Lamiales	153,995	38.00	NC_035233.1	2017
*Salvia miltiorrhiza*	*Lamiaceae*	Lamiales	151,328	38.02	NC_020431.1	2013
*Salvia rosmarinus*	*Lamiaceae*	Lamiales	152,462	38.00	NC_027259.1	2015
*Olea europaea*	*Oleaceae*	Lamiales	155,888	37.80	NC_013707.2	2017
*Castilleja paramensis*	*Orobanchaceae*	Lamiales	152,926	38.20	NC_031805.1	2016
*Pedicularis hallaisanensis*	*Orobanchaceae*	Lamiales	143,469	38.70	NC_037433.1	2018
*Erythranthe lutea*	*Phrymaceae*	Lamiales	153,150	37.70	NC_030212.1	2016
*Digitalis lanata*	*Plantaginaceae*	Lamiales	153,108	38.60	NC_034688.1	2017
*Penstemon fruticosus*	*Plantaginaceae*	Lamiales	152,704	37.90	MG_201976.1	2017
*Plantago depressa*	*Plantaginaceae*	Lamiales	164,617	38.00	NC_041161.1	2019
*Plantago lagopus*	*Plantaginaceae*	Lamiales	145,936	38.30	NC_041420.1	2019
*Plantago maritima*	*Plantaginaceae*	Lamiales	158,358	38.60	NC_028519.1	2015
*Plantago media*	*Plantaginaceae*	Lamiales	164,130	38.00	NC_028520.1	2015
*Plantago ovata*	*Plantaginaceae*	Lamiales	149,739	38.30	NC_041421.1	2019
*Veronica nakaiana*	*Plantaginaceae*	Lamiales	152,319	37.90	NC_031153.1	2016
*Veronica persica*	*Plantaginaceae*	Lamiales	150,198	37.90	NC_031344.1	2016
*Veronicastrum sibericum*	*Plantaginaceae*	Lamiales	152,930	38.30	NC_031345.1	2016
*Scrophularia buergeriana*	*Scrophulariaceae*	Lamiales	153,631	38.00	NC_031437.1	2016
*Scrophularia dentata*	*Scrophulariaceae*	Lamiales	152,600	38.00	NC_036942.1	2018
*Scrophularia henryi*	*Scrophulariaceae*	Lamiales	152,868	38.00	NC_036943.1	2018
*Scrophularia takesimensis*	*Scrophulariaceae*	Lamiales	152,425	38.10	NC_026202.1	2015

### Phylogenetic analysis

For the whole-plastome phylogeny, we aligned the plastome sequences of our six *Penstemon* species, 22 additional species from the Lamiales order, and *So*. *lycopersicum* as an outgroup (Table 1) using the MAFFT webserver [42]. To perform the ML analysis we used the GTR+G4 evolutionary model was performed in IQ-TREE with 1,000 bootstrap support [46, 47]. For the individual gene sequence phylogenies, we made sequence alignments for *ndhF*, *rbcL*, and *rps2* sequences of the same species as the whole-plastome phylogeny using the MUSCLE webserver (https://www.ebi.ac.uk/Tools/msa/muscle/) [48]. For the concatenated phylogeny, we used the aligned sequences of *matK*, *ndhF*, *psaA*, *psbA*, *rbcL*, *rpoC2*, and *rps2* from each species, which we concatenated using Python v2.7.5. We performed ML analyses for each gene sequence and concatenated sequences, using the TN+G4 evolutionary model in IQ-TREE with 1,000 bootstrap support. To view and annotate the optimal ML from all ML analyses, we used the TreeGraph 2 v2.15.0–887 software [49].

## Results and discussion

### Assembly and annotation

The number of paired-end reads we sequenced ranged from 81,521,066 in *P*. *palmeri* to 19,370,452 in *P*. *dissectus* representing an estimated total genome coverage of 18.26x to 6.42x, respectively [5] (Table 2). The mean sequence length for paired reads was 279 bp for all species (Table 2). The NOVOPlasty output log identified between 12,238,056 (*P*. *palmeri*) and 2,414,370 (*P*. *rostriflorus*) reads that aligned to the reference plastome sequence, assembled between 1,141,338 (*P*. *palmeri*) and 643,118 (*P*. *rostriflorus*), at an average organelle coverage of between 62,837 (*P*. *personatus*) and 16,203 (*P*. *cyaneus*) (Table 2). The lengths of each assembled plastome sequences ranged between 152,659 bp (*P*. *palmeri*) and 153,091 (*P*. *personatus*), which were very similar to the previously published *P*. *fruticosus* reference plastome (152,704 bp) [35]. All plastomes showed the typical angiosperm quadripartite structure [50]. The long single copy (LSC) lengths ranged from 84,217 bp (*P*. *personatus*) to 83,795 bp (*P*. *palmeri*). The short single copy (SSC) lengths ranged from 17,818 bp (*P*. *personatus*) to 17,780 bp (*P*. *dissectus*). The IR lengths ranged from 25,564 bp (*P*. *rostriflorus*) to 25,528 bp (*P*. *personatus*) (Table 3).

**Table 2:** Whole genome Illumina (250x250) pair-end sequencing results, and NOVOPlasty *Penstemon* plastome assembly reports.

Whole Genome Sequencing	*P*. *cyaneus*	*P*. *dissectus*	*P*. *palmeri*	*P*. *personatus*	*P*. *rostriflorus*
Nuclear Genome Size (Mbp)^a^	753	462	688	495	565
Number of Reads	40,895,418	19,370,452	81,521,066	33,780,614	36,386,168
Mean Read Length (bp)	314.8	250.7	281.8	256.7	290.8
Sequenced Bases (Gb)	8.54	3.06	13.9	5.44	8.01
Estimated Genome Coverage (x)	10.18	6.42	18.26	10.99	14.42
CG Content (%)	37.62	36.98	36.32	36.72	35.21
**NOVOPlasty Assembly**					
Aligned Reads	9,905,064	3,276,548	12,238,056	6,416,454	2,414,370
Assembled Reads	1,039,070	759,358	1,141,338	1,001,622	643,118
Average Organelle Coverage (x)	16,203	32,084	20,041	62,837	23,605
CG Content (%)	37.86	37.84	37.87	37.87	37.84

^a^Nuclear genome size estimates were produced through flow cytometry by (Broderick et al, 2011).

**Table 3:** Plastome sequence assembly results. Total sizes of the *Penstemon* plastome, long single copy (LSC), short single copy (SSC), and inverted repeat (IR) for each species, as well as the counts of total genes, coding DNA sequence (CDS), rRNA, tRNA, and duplicated genes (within IR regions), followed by the NCBI sequence and annotation submission ID number.

	*P*. *cyaneus*	*P*. *dissectus*	*P*. *palmeri*	*P*. *personatus*	*P*. *rostriflorus*
Total Length	152,822	152,951	152,659	153,091	153,087
LSC	83,940	84,109	83,795	84,217	84,161
SSC	17,812	17,780	17,802	17,818	17,798
IR	25,535	25,531	25,531	25,528	25,564
Total Genes	114	114	114	114	114
CDS	83	83	83	83	83
rRNA	4	4	4	4	4
tRNA	30	30	30	30	30
Duplicated Genes	21	21	21	21	21
NCBI Submission ID	MK391143	MK2183578	MK656967	MK940755	MK940756

In our plastome annotations, we identified identical genes for all species. Each had 83 unique CDS genes and four rRNA genes, as does the *P*. *fruticosus* reference. However, *P*. *fruticosus* has 29 unique tRNA genes and the rest of the plastomes had 30 unique tRNA genes (Table 3). We aligned the five *Penstemon* plastomes to *P*. *fruticosus* and identified 8,577 sequence variants, including 1,729 single nucleotide polymorphisms (SNPs) and 6,848 indels between all species.

We identified possible pseudogenization of the *ndhD* gene in all lineages except *P*. *fruticosus* due to a start-loss missense mutation, which shifted the start codon to the 128^th^ position in the amino acid sequence. The *ndhD* gene in the IR regions had an identical mutation in both IRa and IRb sequences. Additionally, we found at least three indels in the final 18 codons of *ndhD*, which caused frame shifts and early termination of the amino acid sequence. *Penstemon dissectus*, *P*. *palmeri*, and *P*. *cyaneus* had identical *ndhD* gene sequences and indels, which supports the monophyly of this clade as all three taxa inherited this mutation through a common ancestor. We also identified a missense mutation in the stop codon of the *rps12* gene in *P*. *palmeri*, which extended the protein by 22 amino acids. We only observed this mutation in *P*. *palmeri*, which indicates that the mutation occurred after *P*. *palmeri* (subgenus *Penstemon*) diverged from the *P*. *cyaneus* (*Habroanthus*) and *P*. *dissectus* (*Dissecti*) lineages. Without additional taxa sampling from the *Penstemon* and *Habroanthus* subgenera we are unable to determine whether this *rps12* mutation is unique to *P*. *palmeri*, or if it is common in the *Penstemon* subgenus.

### Repeat analysis

Overall, the number and size of repeats were comparable among *Penstemon* species. Our *P*. *fruticosus* reference contained 19 SSRs ranging in size from seven dinucleotide repeats to ten tetranucleotide repeats. The number of SSRs in the species tested were very similar to the reference, ranging from 15 in *P*. *cyaneus* and *P*. *palmeri* to 18 in *P*. *personatus*. *Penstemon dissectus* had the only pentanucleotide repeat (Table 4).

**Table 4:** Simple sequence repeats. The number of each type of simple sequence repeats (SSRs) identified in each plastome using the MISA webserver (http://misaweb.ipk-gatersleben.de/). *Penstemon fruticosus* is included in this study as a reference for the basal clade of *Penstemon*.

	Dinucleotide	Trinucleotide	Tetranucleotide	Pentanucleotide	Total Repeats
*P*. *fruticosus*	7	2	10	0	19
*P*. *cyaneus*	5	1	9	0	15
*P*. *dissectus*	6	1	9	1	17
*P*. *palmeri*	6	1	8	0	15
*P*. *personatus*	7	1	10	0	18
*P*. *rostriflorus*	6	1	10	0	17

Due to their heritable physical location and length, and ease of amplification protocols, SSRs make ideal markers for population genetic and phylogenetic studies of closely related taxa [51]. The majority of identified *Penstemon* plastid SSRs appeared to be homologous loci across the different *Penstemon* lineages. Their exact physical locations and lengths varied due to indel mutations. We observed several incidences where two SSR loci (S1 Table) were physically separated or absent in some linages, but directly adjacent in other taxa. This could indicate that there is an indel mutation between the two loci or point mutations within one or both SSR intervening sequence(s). These loci could be useful to test homology and observe changes in populations, lineages, and clades over time. Upon validation of universal primers for physical location, SSR length, and point mutations, these SSRs could be excellent population genetics tools, but such evaluations were beyond the scope of this research.

**Fig 2:**
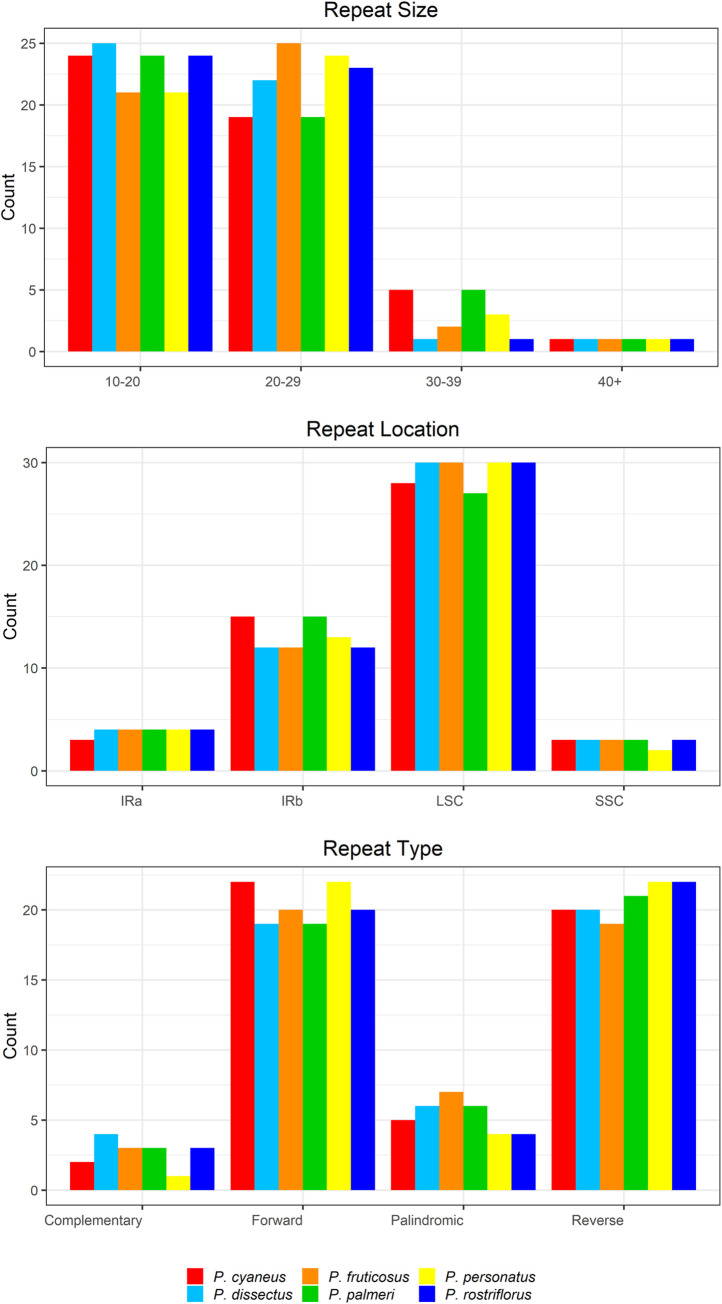
Chloroplast sequence repeats. Repeat size, location, and type for all six *Penstemon* subgenera identified using REPuter.

REPuter identified 49 total repeats (reverse, complement, forward, and palindrome) for all species including *P*. *fruticosus*. However, the types, sizes, and locations of these repeats varied among species (Fig 2). The majority of repeats (88–96%) were smaller than 30 bp for all species and were located in the LSC region (61–55%). *Penstemon fruticosus* had the most palindromic repeats (14%) and *P*. *personatus* and *P*. *rostriflorus* had the fewest (8%). *Penstemon dissectus* had the most complimentary repeats (8%) and *P*. *personatus* had the fewest (2%).

The ploidy level within the *Penstemon* genus ranges from diploid to dodecaploid, with diploid genome sizes ranging from 463 Mbp in *P*. *dissectus* to 922 Mbp in *P*. *nitidus* [5]. Although the species included in this study are diploid, *P*. *cyaneus* has a nuclear genome up to 63% larger than the other taxa in this study (Table 2). Along with its large nuclear genome size, *P*. *cyaneus* has approximately double the number of repetitive elements in its nuclear genome as compared to *P*. *dissectus* and *P*. *fruticosus* [52]. The causes and origins of diploid genome enlargement in *Penstemon* remains mostly unstudied, and it is unknown whether gene duplication including ectopic recombination, replication slippage, or retrotransposition also play a role. Since plastome size is uninfluenced by ploidy level or nuclear genome size, the number and composition of the repetitive elements in the chloroplasts are comparable between our *Penstemon* taxa. All plastome genes in a given lineage are orthologous, inherited from a common maternal ancestor, which make plastomes ideal for phylogenetic studies as determining homologs, paralogs, and pseudogenization unnecessary. Investigations of nuclear genome mutations are ideal for studying the genetic changes that occurred during speciation and diversification, but plastomes are ideal to construct maternal evolutionary histories and phylogenies.

### Codon usage

The RSCU for each amino acid was nearly identical among all species (S2 Table). We observed 61 unique codons for the 20 amino acids used in all CDS in our *Penstemon* plastomes. Seventeen of these codons were preferred over the other codons for the same amino acid. However, our plastome annotations only identified 30 tRNA codon genes in our *Penstemon* species (29 in *P*. *fruticosus*), only 11 of which were for the preferred amino acid codons. This indicates that the remaining 31 tRNA codons, including five preferred tRNA codons, come directly from the host cell’s cytosol and are not produced by the chloroplast’s genome. Chloroplast genomes commonly encode around 30 tRNA genes, and like mitochondria, import tRNAs from the cytosol which are produced by the nucleus [53]. The overall chloroplast genome size is greatly reduced from the ancestral cyanobacteria species, which have up to 12,000 CDS genes, compared to a chloroplast’s 80–230, primarily through horizontal gene transfer [54, 55].

### Synteny block analyses

**Fig 3:**
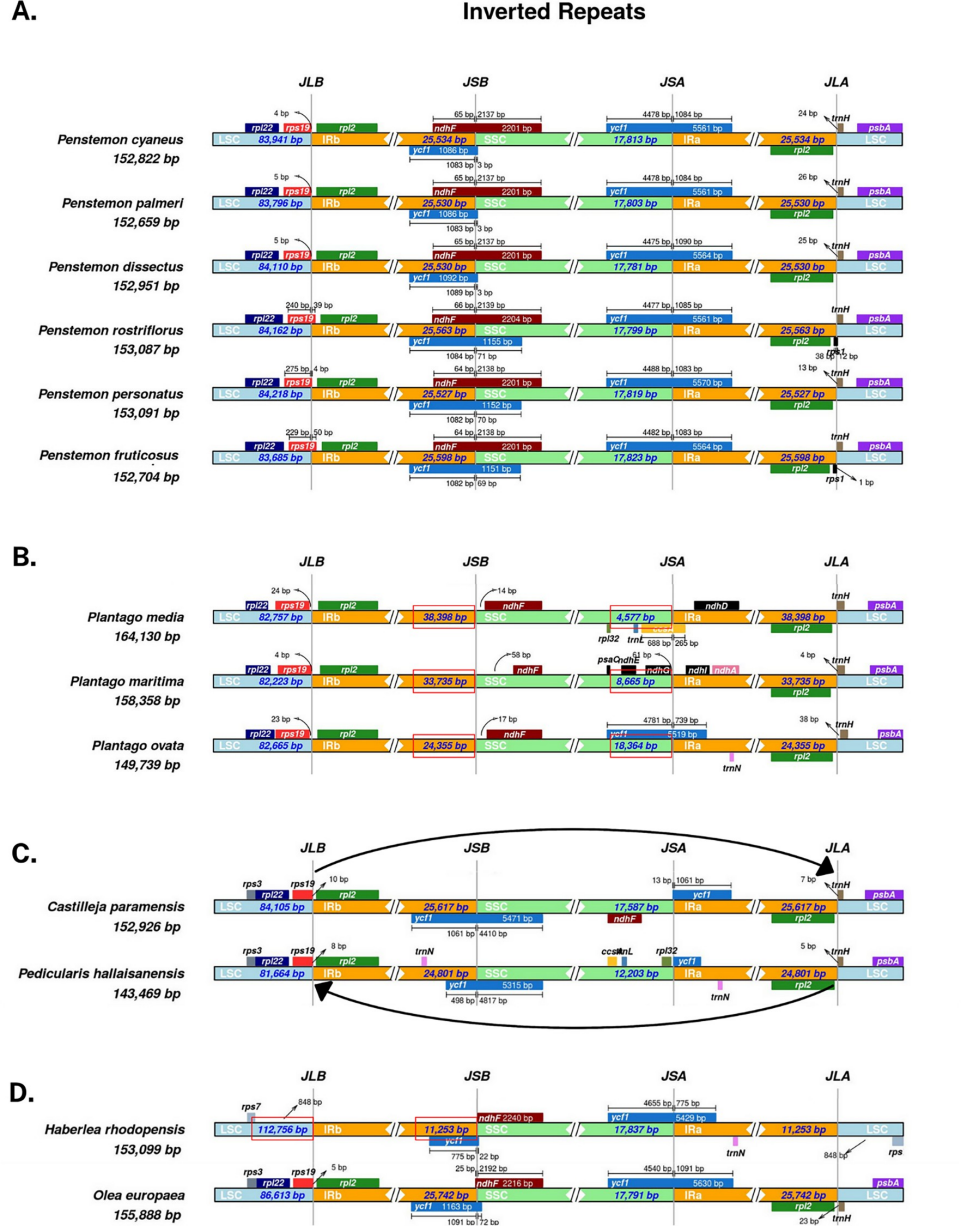
IRScope visualizations of inverted repeat (IR) and short-single copy (SSC) junctions. A. Variation among *Penstemon* taxa: the *ycf1* fragment at the SSC-IRb junction (JSB) in *P*. *dissectus*, *P*. *palmeri*, and *P*. *cyaneus*. B. Successive expansions of the IR regions into the SSC region observed in the genus *Plantago*. C. Total inversion of the IRa-SSC-IRb plastome segment in the Orobanchaceae genera *C*. *paramensis* and *P*. *hallaisanensis*. D. A reduction in IR length associated with an expansion of the LSC region in *H*. *rhodopensis* compared to our basal lineage, *Olea europaea*.

The IR junctions were well conserved within the *Penstemon* genus as we only observed minor expansions/contractions that were not useful to clarify phylogenetic relationships (Fig 3A). *Penstemon fruticosus* had the largest IR regions at 25,598 bp and *P*. *personatus* had the shortest at 25,527 bp. However, we observed several structural variants within the Lamiales order that did clarify phylogeny. We observed major structural changes within the Plantaginaceae family, specifically several expansions of the IR regions into the SSC regions within the genus *Plantago* (Fig 3B). The sizes of the IR region were highly variable, ranging from 20,336 bp, in *Pl*. *lagopus*, to 38,398 bp in *Pl*. *media*. The remaining taxa in this family, excluding those within *Plantago*, have consistent IR region sizes of 25,465 bp to 25,757 bp. One of the most phylogenetically significant structural changes we observed was the complete inversion of the IRb-SSC-IRa regions in the Orobanchaceae taxa *Castilleja paramensis* and *Pedicularis hallaisanensis* (Fig 3C). This inversion has major implications for the placement of this family within the phylogeny of the Lamiales order, which we will discuss in the context of our ML phylogenetic analyses. We also observed the expansion of the LSC region and contraction of the IR regions in *Haberlea rhodopensis* (Fig 3D).

Although IRScope is a visualization tool and not an analytical tool, it is demonstrably useful for observing and identifying plastome structural, or synteny block, changes near the IR junctions such as inversions, and IR expansion/contraction. These types of structural have previously been identified in plastids of the Orobanchaceae family [14] and in the genera *Pelargonium* [56] and *Plantago* [57]. Plastome structural variants are heritable and can provide valuable insight into evolution and speciation [58]. Many of these mutations can be traced through the evolutionary process of land plants and used as a phylogenetic tool to provide evidence of shared ancestry [59, 60]. We found that the major structural mutations we observed with IRScope correlated with highly supported clades in our whole-plastome phylogenetic analysis. Unfortunately, these informative mutations are excluded from phylogenetic analyses that use isolated gene sequences, both individual and concatenated, as they are absent of genome structural variants.

### Phylogenetic analyses

**Fig 4:**
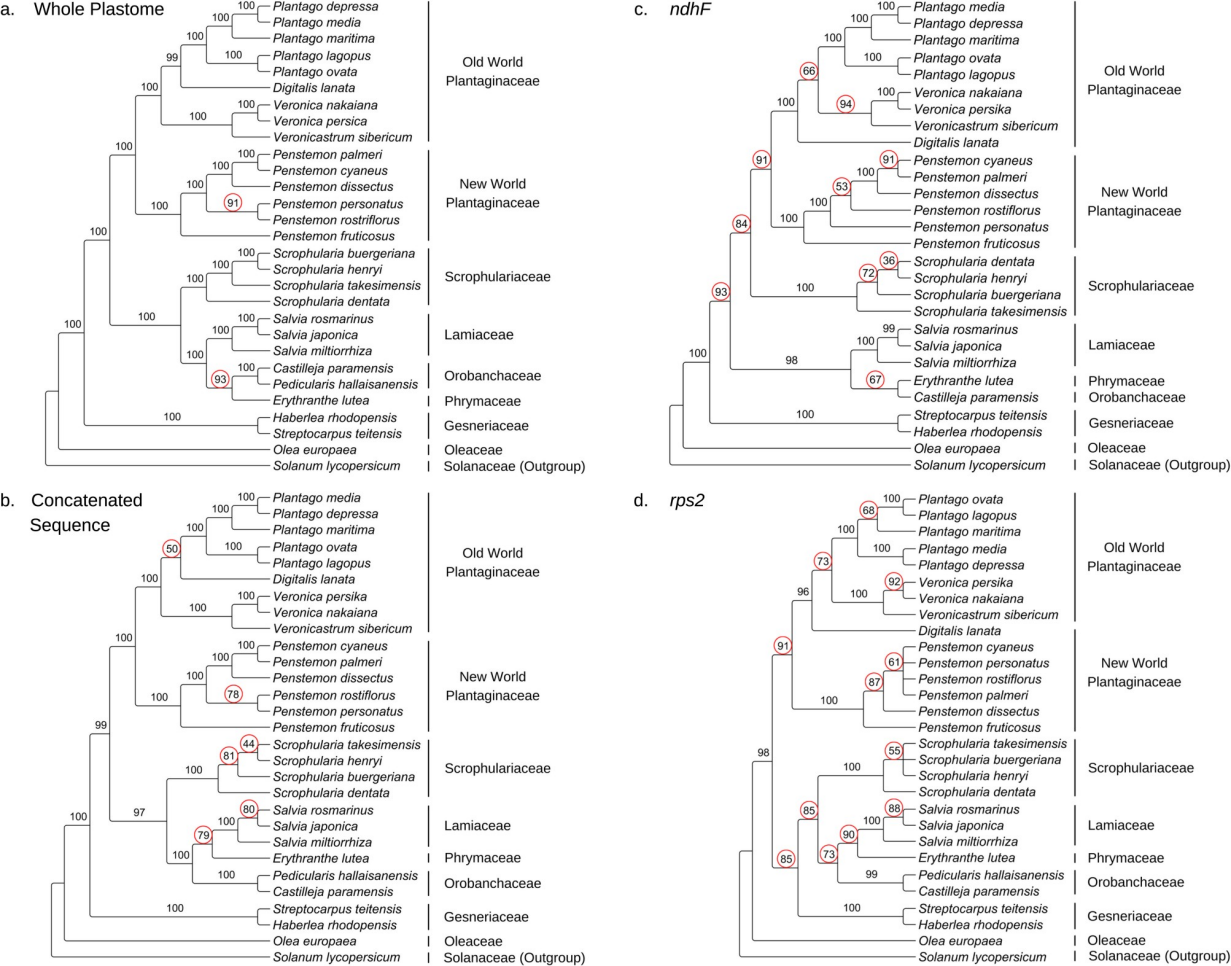
Maximum likelihood phylogenies of the Whole-plastome, concatenated sequence, *ndhF*, and *rps2* sequences. A. The whole-plastome sequence phylogeny. B. The concatenated sequence (*matK*, *ndhF*, *psaA*, *psbA*, *rbcL*, *rpoC2*, and *rps2*) phylogeny. C. The *ndhF* sequence phylogeny. D. The *rps2* sequence phylogeny. Bootstrap support values below 95 are emphasized with red circles.

Our whole-plastome phylogenetic tree supports the revision of the Scrophulariaceae. In previous studies only the basal nodes have consistently had a high degree of resolution and nodal support [14]. While lineages inclusive of more recent diversification, such as observed in *Penstemon* [2] and *Plantago* [16], typically have poor resolution and low nodal support (polyphyly, paraphyly, and polytomies) and are typified by low levels of variation in individual gene sequences [17]. These lineages are only now well resolved in our whole-plastome phylogeny with all but two nodes having statistically significant bootstrap support values (BSV) above 95 [61], and all nodes with BSV above 90 (Fig 4A). This high overall nodal support is an indication of a highly stable and reliable phylogeny [62].

The phylogenetic trees derived from individual gene sequences in this study had drastically different topologies with lower overall nodal support as compared to our whole-plastome phylogeny. In our whole-plastome phylogeny, we observed 24 of 27 nodes with BSV of 100 (Table 5). In contrast, the phylogenies based on the individual gene sequences (*matK*, *ndhF*, *psaA*, *psbA*, *rbcL*, *rpoC2*, and *rps2*) and concatenated sequence observed nodes with BS of 100 ranging from two (*psbA*) to 18 (concatenated). Although the phylogeny based on concatenated sequences (Fig 4B) had greater nodal support than the phylogenies base on individual sequences, 22% of its nodes were not statistically significant with BSV less than 90. Only two (7%) of the nodes in the whole-plastome phylogeny were not significant, and no nodes had BS less than 90.

**Table 5:** Comparison of nodal support in our plastid phylogenetic trees. The node count and percentages for each phylogeny based on whole-plastome, individual gene sequences (*matK*, *ndhF*, *psaA*, *psbA*, *rbcL*, *rpoC2*, and *rps2*), and concatenated sequences with the specified level of nodal support.

Sequence	Total Nodes	BSV = 100	BSV ≤ 95	BSV > 95	BSV > 90
Whole-Plastome	27	24 (89%)	25 (93%)	2 (7%)	0 (0%)
Concatenated	27	18 (67%)	20 (74%)	6 (22%)	6 (22%)
*matK*	26	14 (54%)	17 (65%)	10 (38%)	9 (35%)
*ndhF*	25	13 (52%)	15 (60%)	10 (40%)	6 (24%)
*psaA*	26	8 (31%)	12 (46%)	14 (54%)	11 (42%)
*psbA*	26	2 (8%)	9 (35%)	17 (65%)	14 (54%)
*rbcL*	26	8 (31%)	11 (42%)	15 (58%)	12 (46%)
*rpoC2*	26	14 (54%)	16 (62%)	10 (38%)	6 (23%)
*rps2*	22	6 (27%)	11 (50%)	11 (50%)	7 (32%)

The sequences selected for phylogenetic analysis is crucial to the inference of the resulting tree. Genes that are vital to the function of photosynthesis such as *psaA* and *psbA*, for example, are highly conserved since mutations in these encoded proteins must not be deleterious to the proper function of photosynthesis. Our ML phylogeny of these two gene sequences had very low resolution because there was very little variation between species (S1 Fig). We also observed polytomic clades within the genera *Penstemon* and *Scrophularia* due to little variation between species (Fig 4D). Sequence concatenation appears to stabilize resolution and nodal support as the number of sequences in the concatenation grows. However, the resolution is unreliable because concatenation creates an artificial chromosome sequence constructed from a subset of gene sequences in an arbitrary order. Even if all CDS sequences are used, it will still be unreliable and will omit heritable structural variants including unannotated pseudogenes that are crucial to constructing the evolutionary history of a lineage.

The origin of the complete inversion of the IRa-SSC-IRb region we observed in *Castilleja paramensis* and *Ped*. *hallaisanensis* with IRScope (Fig 3C) is a crucial structural variant that may play a key role in understanding the evolution and diversification of Orobanchaceae. However, the locus for the *ndhF* gene is located within this inversion but is unannotated in *Ped*. *hallaisanensis* due to a deletion or pseudogenization, which causes its sequence to be omitted from phylogenetic analyses of the *ndhF* sequence alone or in concatenation studies (Fig 4C). The placement of this family varies greatly in each phylogenetic analysis we performed. Interestingly, the Orobanchaceae with this inversion is often placed as a basal clade to most of the Lamiales after *O*. *europaea* in the *rbcL* phylogeny (S2 Fig), or to the Phrymaceae and Lamiaceae families in the *psaA* (S1 Fig), *psbA* (S1 Fig), *rpoC2* (S2 Fig), *rps2* (Fig 4D), and concatenated sequence phylogeny (Fig 4B). The nodal support of this family varied from very low in the *psbA* phylogeny (BS = 7) to significant (BS≤95) in the whole-plastome, concatenated sequence, and *ndhF* phylogenies.

Our method of isolating and extracting plastome sequences from whole-genome sequencing data without additional DNA extraction steps is a cost-efficient method for plastome sequencing and assembly. Whole genome sequencing on the Illumina HiSeq 2500 (2x250) platform can produce 125–150 Gb on a single flow cell lane, with multiplexing possible up to 12 samples, at an approximate cost of $20.00–30.00 per Gb of data. NOVOPlasty recovered 16,000x to 62,000x coverage from our whole genome sequencing runs, indicating that minimal whole genome coverage (Table 2) will contain sufficient plastome sequences for assembly and analysis.

## Conclusion

As a result of this research, we have produced and submitted the assembled and annotated plastome sequences of *P*. *cyaneus*, *P*. *dissectus*, *P*. *palmeri*, *P*. *personatus*, *and P*. *rostriflorus* to the NCBI GenBank DNA sequence database. These sequences, with the previously published *P*. *fruticosus* plastome [35], complete the representation of all *Penstemon* subgenera.

Whole-plastome based phylogenetic analyses improved the resolution of the Lamiales order, the Plantaginaceae, and the genus *Penstemon* with high nodal support. Whole-plastome phylogenies are superior to both individual and concatenated chloroplast sequences as they provide more polymorphic markers that add statistical power to the tested hypotheses [34], they provide high statistical nodal support, and they detect heritable genome rearrangements, including inversions and IR expansions/contractions, and group taxa according to these genome structural changes. We found that a major limitation of both individual and concatenated gene sequence-based phylogenies is that heritable structural rearrangements are excluded from the analyses. Since these rearrangements are heritable, they are crucial to accurate phylogenetic relationships and may be critical to the resolution of phylogenetic ambiguities of closely related and recently classified taxa [16, 63].

Our findings also suggest that *Penstemon* represents a unique monophyletic lineage in the Plantaginaceae family and warrant further exploration with a broad sampling of *Penstemon* taxa along with Old World and New World genera of the Plantaginaceae family to resolve problematic clades; a process particularly challenging using conventional markers and methods. Such work would assuredly further our understanding of the origins, evolution, and diversification of *Penstemon* within Plantaginaceae.

## Supporting information

S1 Fig Maximum likelihood phylogenies of the whole-plastome, *matK*, *psaA*, and *psbA* sequences. A. The whole-plastome sequence phylogeny. B. The *matK* sequence phylogeny. C. The *psaA* sequence phylogeny. D. The *psbA* sequence phylogeny. Bootstrap values below 95 are emphasized with red circles.
(TIF)

S2 Fig Maximum likelihood phylogenies of the whole-plastome, *rbcL*, and *rpoC2* sequences. A. The whole-plastome sequence phylogeny. B. The *rbcL* sequence phylogeny. C. The *rpoC2* sequence phylogeny. Bootstrap values below 95 are emphasized with red circles.
(TIF)

S1 Table Simple sequence repeats (SSR) by location in each *Penstemon* plastome. The physical locations and lengths of each SSR identified using MISA. Sizes and positions of each SSR varies between taxa due to indel mutations. We observed several incidences where two SSR loci were physically separated or absent in some linages, but directly adjacent in other taxa (bold text).
(DOCX)

S2 Table Relative synonymous codon usage (RSCU) in each *Penstemon* plastome. Codons with moderate to high preference, RSCU values above 1.2, for each amino acid are in bold text. Only leucine, arginine, and serine have more than one codon with high preferences. Most amino acids with two codons had a preference for one codon, the exceptions being cysteine, lysine, and asparagine.
(DOCX)
