# Supplementary figures and images for: Improving phylogenetic resolution of the Lamiales using the complete plastome sequences of six Penstemon species
Source: PLoS One. 2021 Dec 15;16(12):e0261143. doi: 10.1371/journal.pone.0261143 (PMC8673674; doi:10.1371/journal.pone.0261143)

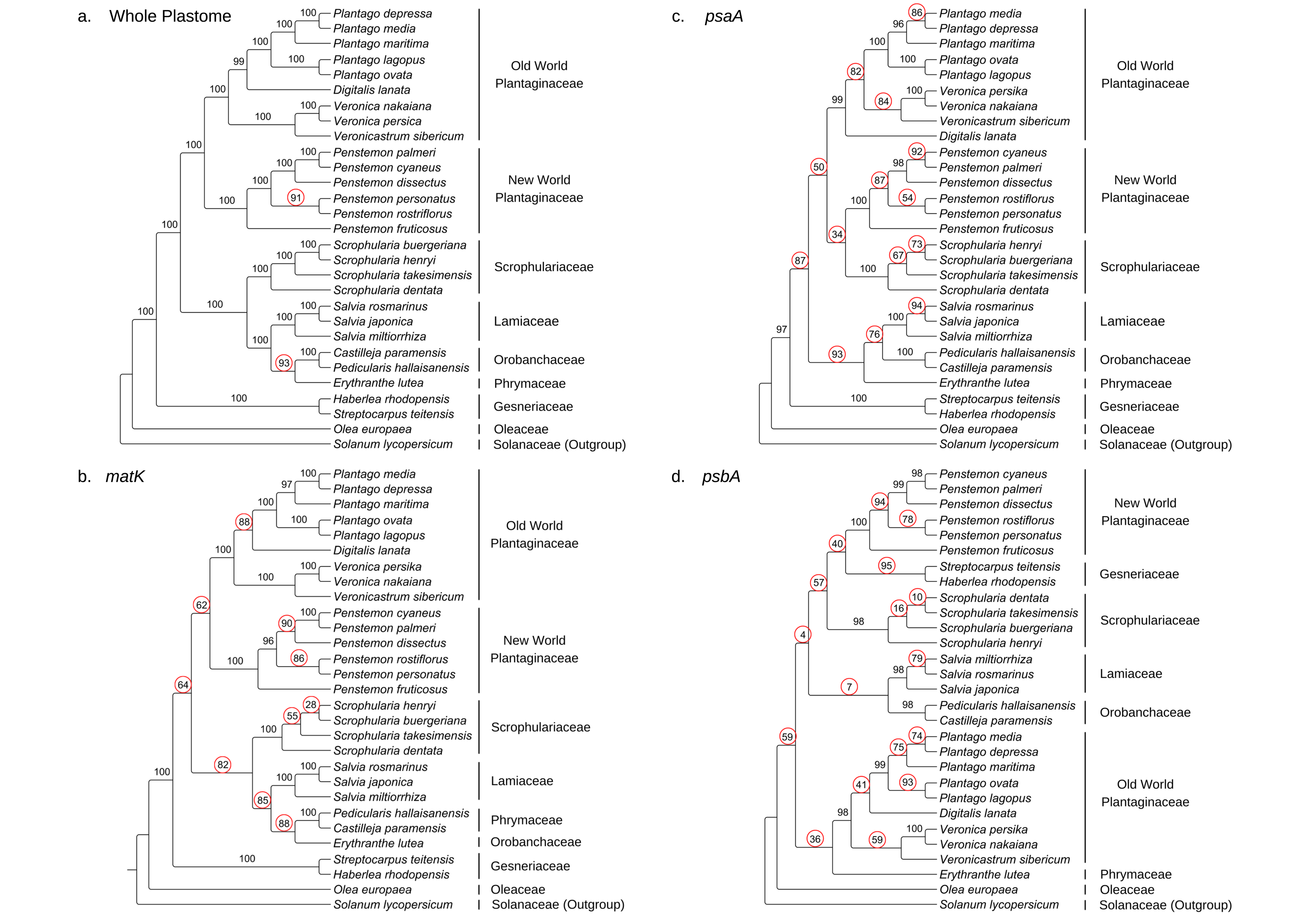

Supplement: S1 Fig — A. The whole-plastome sequence phylogeny. B. The matK sequence phylogeny. C. The psaA sequence phylogeny. D. The psbA sequence phylogeny. Bootstrap values below 95 are emphasized with red circles. (TIF) [file pone.0261143.s001.tif]

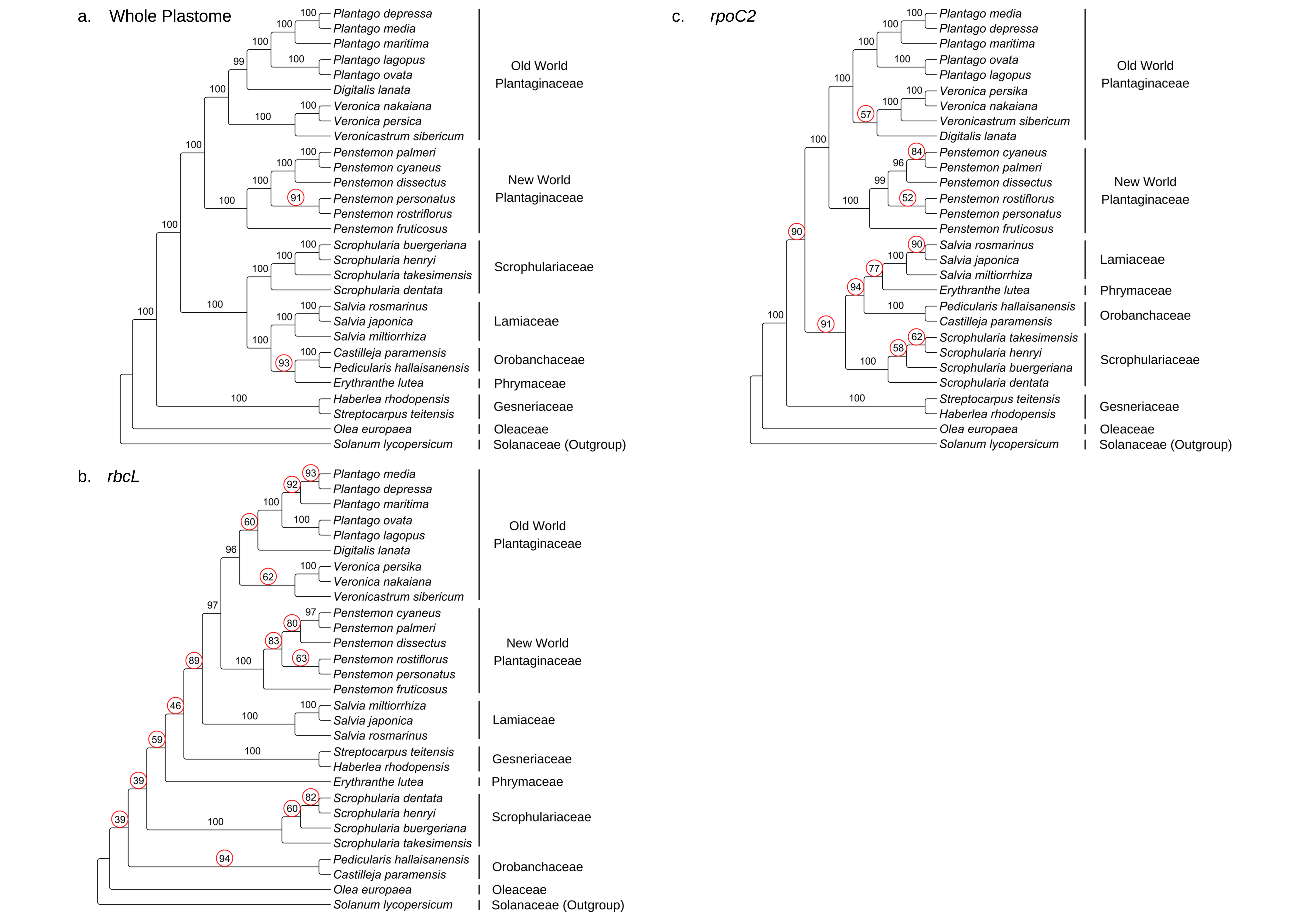

Supplement: S2 Fig — A. The whole-plastome sequence phylogeny. B. The rbcL sequence phylogeny. C. The rpoC2 sequence phylogeny. Bootstrap values below 95 are emphasized with red circles. (TIF) [file pone.0261143.s002.tif]
