# Supplementary material for: Improving phylogenetic resolution of the Lamiales using the complete plastome sequences of six Penstemon species
Source: PLoS One. 2021 Dec 15;16(12):e0261143. doi: 10.1371/journal.pone.0261143 (PMC8673674; doi:10.1371/journal.pone.0261143)
Supplement: S1 Table — The physical locations and lengths of each SSR identified using MISA. Sizes and positions of each SSR varies between taxa due to indel mutations. We observed several incidences where two SSR loci were physically separated or absent in some linages, but directly adjacent in other taxa (bold text). (DOCX) [file pone.0261143.s003.docx]

**S1 Table. Simple sequence repeats (SSR) by location in each *Penstemon* plastome.** The physical locations and lengths of each SSR identified using MISA. Sizes and positions of each SSR varies between taxa due to indel mutations. We observed several incidences where two SSR loci were physically separated or absent in some linages, but directly adjacent in other taxa (bold text).

| **SSR Sequence** | ***Penstemon fruticosus*** | | ***Penstemon cyaneus*** | | ***Penstemon dissectus*** | | ***Penstemon palmeri*** | | ***Penstemon personatus*** | | ***Penstemon rostriflorus*** | |
| --- | --- | --- | --- | --- | --- | --- | --- | --- | --- | --- | --- | --- |
|  | **Size** | **Location** | **Size** | **Location** | **Size** | **Location** | **Size** | **Location** | **Size** | **Location** | **Size** | **Location** |
| A | - | - | 10 | 2170 - 2179 | - | - | 10 | 2171 - 2180 | - | - | - | - |
| T | 14 | 2256 - 2269 | - | - | 11 | 2259 - 2269 | - | - | 13 | 2361 - 2373 | 14 | 2361 - 2374 |
| T | 12 | 2363 - 2374 | 15 | 2372 - 2386 | 15 | 2363 - 2377 | 15 | 2363 - 2377 | 12 | 2925 - 2936 | 16 | 2926 - 2941 |
| T | - | - | 12 | 2943 - 2954 | 13 | 2929 - 2941 | 12 | 2930 - 2941 | - | - | - | - |
| T | - | - | - | - | - | - | 10 | 4085 - 4094 | - | - | - | - |
| T | 11 | 4322 - 4332 | 10 | 4336 - 4345 | 12 | 4324 - 4335 | 11 | 4329 - 4339 | 12 | 4313 - 4324 | - | - |
| T | - | - | - | - | - | - | - | - | 10 | 4352 - 4361 | 10 | 4360 - 4369 |
| T | 11 | 8369 - 8379 | 11 | 8388 - 8398 | 11 | 8370 - 8380 | 11 | 8375 - 8385 | 11 | 8353 - 8363 | 11 | 8406 - 8416 |
| AT | 5 | 9736 - 9745 | 5 | 9755 - 9764 | 5 | 9737 - 9746 | 5 | 9742 - 9751 | 5 | 9720 - 9729 | 5 | 9773 - 9782 |
| AT | 6 | 17176 - 17187 | 5 | 17207 - 17216 | 5 | 17171 - 17180 | 5 | 17188 - 17197 | 5 | 17161 - 17170 | - | - |
| TGAC | 3 | 19407 - 19418 | 10 | 20921 - 20930 | 3 | 19271 - 19282 | - | - | 3 | 19336 - 19347 | 3 | 19398 - 19409 |
| A | - | - | - | - | 10 | 19913 - 19922 | 10 | 19804 - 19813 | 10 | 19834 - 19843 | - | - |
| TA | - | - | - | - | - | - | 5 | 20895 - 20904 | - | - | - | - |
| T | 12 | 21271 - 21282 | 10 | 21308 - 21317 | 14 | 21136 - 21149 | 11 | 21021 - 21031 | 10 | 21204 - 21213 | 10 | 21275 - 21284 |
| T | 15 | 21671 - 21685 | - | - | - | - | - | - | 13 | 21216 - 21228 | 11 | 21663 - 21673 |
| TCTA | 3 | 21860 - 21871 | 3 | 21492 - 21503 | 3 | 21717 - 21728 | 3 | 21592 - 21603 | 3 | 21790 - 21801 | 3 | 21848 - 21859 |
| A | - | - | - | - | - | - | 11 | 21907 - 21917 | - | - | - | - |
| **A** | 12 | 25966 - 25977 | - | - | 11 | 25822 - 25832 | - | - | - | - | - | - |
| **A + C** | - | - | - | - | - | - | - | - | 14+10 | 25887 - 25910 |  |  |
| **C** | - | - | - | - | - | - | - | - | - | - | 11 | 25938 - 25948 |
| TGA | 4 | 31860 - 31871 | - | - | - | - | - | - | - | - | - | - |
| TA | 5 | 31950 - 31959 | 5 | 31577 - 31586 | 5 | 31805 - 31814 | 5 | 31689 - 31698 | 7 | 31883 - 31896 | 5 | 31934 - 31943 |
| A | 10 | 32056 - 32065 | 13 | 31693 - 31705 | 12 | 31921 - 31932 | 12 | 31805 - 31816 | - | - | 10 | 32050 - 32059 |
| TA | 5 | 32251 - 32260 | 5 | 31894 - 31903 | 5 | 32121 - 32130 | 5 | 32006 - 32015 | 5 | 32200 - 32209 | 5 | 32248 - 32257 |
| T | 11 | 33166 - 33176 | 11 | 32807 - 32817 | 13 | 33035 - 33047 | 12 | 32919 - 32930 | 12 | 33114 - 33125 | 11 | 33163 - 33173 |
| A | 10 | 34361 - 34370 | - | - | - | - | - | - | - | - | - | - |
| T | 10 | 36190 - 36199 | 12 | 36474 - 36485 | 10 | 36072 - 36081 | 14 | 35747 - 35760 | - | - | 10 | 36189 - 36198 |
| TA | 5 | 36358 - 36367 | - | - | 5 | 36235 - 36244 | - | - | 5 | 36316 - 36325 | 5 | 36362 - 36371 |
| T | - | - | - | - | 15 | 36702 - 36716 | 14 | 36374 - 36387 | 14 | 36790 - 36803 | 10 | 36829 - 36838 |
| A | - | - | - | - | 10 | 38369 - 38378 | - | - | - | - | - | - |
| TTAG | 3 | 40283 - 40294 | 4 | 40409 - 40424 | 5 | 40642 - 40661 | 4 | 40311 - 40326 | 4 | 40727 - 40742 | 4 | 40756 - 40771 |
| T | 10 | 44070 - 44079 | 10 | 44183 - 44192 | 10 | 44416 - 44425 | 10 | 44085 - 44094 | 10 | 44500 - 44509 | 10 | 44528 - 44537 |
| ATA | 4 | 44124 - 44135 | - | - | - | - | - | - | - | - | - | - |
| AT | 5 | 49488 - 49497 | 5 | 49631 - 49640 | 5 | 49876 - 49885 | 5 | 49534 - 49543 | 5 | 49959 - 49968 | 5 | 49978 - 49987 |
| TAACT | - | - | - | - | 3 | 50695 - 50709 | - | - | - | - | - | - |
| GAAA | 3 | 50479 - 50490 | 3 | 50622 - 50633 | 3 | 50872 - 50883 | 3 | 50525 - 50536 | 3 | 50951 - 50962 | 3 | 50970 - 50981 |
| AATA | 4 | 58725 - 58740 | 3 | 57918 - 57929 | 3 | 58162 - 58173 | 3 | 57819 - 57830 | 3 | 58234 - 58245 | 3 | 58256 - 58267 |
| T | 12 | 58892 - 58903 | 11 | 59019 - 59029 | 10 | 59263 - 59272 | 11 | 58920 - 58930 | 10 | 59335 - 59344 | 11 | 59357 - 59367 |
| T | 12 | 59260 - 59271 | 13 | 59386 - 59398 | 14 | 59629 - 59642 | 15 | 59287 - 59301 | 12 | 59701 - 59712 | 13 | 59719 - 59731 |
| A | 10 | 60483 - 60492 | - | - | - | - | - | - | 10 | 60522 - 60531 | - | - |
| **T** | - | - | 12 | 63873 - 63884 | - | - | 11 | 63775 - 63785 | - | - | - | - |
| **T + G** | 10+13 | 63726 - 63748 |  |  | 10+11 | 64116 - 64136 | - | - | - | - | 12+12 | 64190 - 64213 |
| **G** |  |  |  |  | - | - | - | - | 12 | 64198 - 64209 | - | - |
| TTTC |  |  | 3 | 64759 - 64770 | - | - | - | - | - | - | - | - |
| T | 13 | 64864 - 64876 | - | - | - | - | - | - | - | - | - | - |
| A | - | - | - | - | - | - | - | - | - | - | 10 | 65308 - 65317 |
| T | - | - | 10 | 67795 - 67804 | - | - | 10 | 67697 - 67706 | - | - | - | - |
| **TTC** | - | - | 4 | 70399 - 70425 | - | - | - | - | - | - | 4 | 70691 - 70702 |
| **TTC + T** | - | - | - | - | 4+11 | 70635 - 70657 | 4+12 | 70301 - 70324 | 4+12 | 70692 - 70715 | - | - |
| A | - | - | 10 | 71634 - 71643 | 10 | 71865 - 71874 | 10 | 71534 - 71543 | 11 | 71924 - 71934 | 11 | 71920 - 71930 |
| TTTC | 3 | 71895 - 71906 | 3 | 72078 - 72089 | 3 | 72294 - 72305 | 3 | 71978 - 71989 | 3 | 72369 - 72380 | 3 | 72350 - 72361 |
| A | 11 | 79171 - 79181 | 11 | 79350 - 79360 | 11 | 79560 - 79570 | 10 | 72058 - 72067 | - | - | - | - |
| A | - | - | - | - | - | - | 11 | 79243 - 79253 | 11 | 79633 - 79643 | 11 | 79614 - 79624 |
| T | - | - | 10 | 97299 - 97308 | 10 | 97500 - 97509 | 10 | 97189 - 97198 | - | - | - | - |
| TAAA | - | - | 3 | 102924 - 102935 | 3 | 103106 - 103117 | 3 | 102814 - 102825 | 3 | 103197 - 103208 | 3 | 103179 - 103190 |
| A | - | - | 11 | 103392 - 103402 | 17 | 103568 - 103584 | - | - | 10 | 103668 - 103677 | - | - |
| T | - | - | 11 | 105166 - 105176 | 11 | 105349 - 105359 | 11 | 105054 - 105064 | 11 | 105442 - 105452 | 11 | 105435 - 105445 |
| A | 10 | 107426 - 107435 | 10 | 107747 - 107756 | 10 | 107930 - 107939 | - | - | 11 | 108023 - 108033 | - | - |
| TATC | 3 | 109805 - 109816 | - | - | - | - | - | - | - | - | - | - |
| T | - | - | 10 | 109614 - 109623 | - | - | - | - | - | - | - | - |
| T | 14 | 114221 - 114234 | 14 | 114396 - 114409 | 14 | 114574 - 114587 | 14 | 114276 - 114289 | 14 | 114662 - 114675 | 14 | 114635 - 114648 |
| AATT | 3 | 114236 - 114247 | 3 | 114411 - 114422 | 3 | 114589 - 114600 | 3 | 114291 - 114302 | 3 | 114677 - 114688 | 3 | 114650 - 114661 |
| T | 11 | 114737 - 114747 | 11 | 114848 - 114858 | - | - | 11 | 114728 - 114738 | - | - | - | - |
| T | - | - | 11 | 114912 - 114922 | 11 | 115090 - 115100 | 11 | 114792 - 114802 | 11 | 115184 - 115194 | 11 | 115151 - 115161 |
| A | 10 | 114825 - 114834 | 10 | 115000 - 115009 | 10 | 115178 - 115187 | 10 | 114880 - 114889 | 10 | 115272 - 115281 | 10 | 115239 - 115248 |
| T | 10 | 115260 - 115269 | - | - | - | - | 10 | 115315 - 115324 | - | - | - | - |
| T | 10 | 115319 - 115328 | - | - | - | - | 11 | 115374 - 115384 | - | - | - | - |
| T | 12 | 115437 - 115448 | 10 | 115435 - 115444 | - | - | - | - | - | - | - | - |
| T | - | - | 11 | 115494 - 115504 | 10 | 115613 - 115622 | - | - | 10 | 115707 - 115716 | 10 | 115674 - 115683 |
| T | - | - | - | - | 11 | 115672 - 115682 | - | - | 10 | 115766 - 115775 | 10 | 115733 - 115742 |
| T | - | - | - | - | 12 | 115790 - 115801 | - | - | 12 | 115884 - 115895 | - | - |
| A | - | - | 10 | 119473 - 119482 | 10 | 119654 - 119663 | 10 | 119353 - 119362 | - | - | - | - |
| T | 11 | 137248 - 137258 | 11 | 137421 - 137431 | 11 | 137593 - 137603 | 11 | 137298 - 137308 | 11 | 137684 - 137694 | 11 | 137647 - 137657 |
| T | 12 | 145812 - 145823 | 10 | 144706 - 144715 | 12 | 146085 - 146096 | 10 | 144580 - 144589 | - | - | 10 | 144960 - 144969 |
| T | - | - | 12 | 145922 - 145933 | - | - | 12 | 145796 - 145807 | 12 | 146178 - 146189 | 12 | 146185 - 146196 |
| G | 10 | 147511 - 147520 | - | - | - | - | 11 | 147488 - 147498 | 10 | 147870 - 147879 | 12 | 147875 - 147886 |
| GAAA | - | - | 3 | 147617 - 147627 | - | - | - | - | - | - | - | - |
| GAAA | 3 | 148020 - 148031 | 3 | 148128 - 148139 | 3 | 148288 - 148299 | 3 | 148000 - 148011 | 3 | 148381 - 148392 | 3 | 148388 - 148399 |
| TA | 5 | 149477 - 149486 | - | - | - | - | - | - | - | - | - | - |
| AT | - | - | - | - | - | - | - | - | 5 | 149235 - 149244 | 5 | 149244 - 149253 |
| T | - | - | - | - | - | - | - | - | 11 | 150943 - 150953 | 10 | 150950 - 150959 |
| TTTA | 3 | 150784 - 150795 | - | - | - | - | - | - | 3 | 151138 - 151149 | 3 | 151144 - 151155 |
| A | 11 | 150817 - 150827 | 11 | 150914 - 150924 | 12 | 151059 - 151070 | 10 | 150778 - 150787 | - | - | - | - |
| A | - | - | 10 | 151196 - 151205 | - | - | - | - | 12 | 151171 - 151182 | 10 | 151177 - 151186 |
| T | - | - | - | - | 10 | 151439 - 151448 | - | - | - | - | - | - |
| T | 10 | 152361 - 152370 | 11 | 152477 - 152487 | 10 | 152608 - 152617 | - | - | 11 | 152747 - 152757 | 10 | 152728 - 152737 |
