# Supplementary material for: Improving phylogenetic resolution of the Lamiales using the complete plastome sequences of six Penstemon species
Source: PLoS One. 2021 Dec 15;16(12):e0261143. doi: 10.1371/journal.pone.0261143 (PMC8673674; doi:10.1371/journal.pone.0261143)
Supplement: S2 Table — Codons with moderate to high preference, RSCU values above 1.2, for each amino acid are in bold text. Only leucine, arginine, and serine have more than one codon with high preferences. Most amino acids with two codons had a preference for one codon, the exceptions being cysteine, lysine, and asparagine. (DOCX) [file pone.0261143.s004.docx]

**S2 Table. Relative synonymous codon usage (RSCU) in each *Penstemon* plastome.** Codons with moderate to high preference, RSCU values above 1.2, for each amino acid are in bold text. Only leucine, arginine, and serine have more than one codon with high preferences. Most amino acids with two codons had a preference for one codon, the exceptions being cysteine, lysine, and asparagine.

|  |  | *P. fruticosus* | | *P. cyaneus* | | *P. dissectus* | | *P. palmeri* | | *P. personatus* | | *P. rostriflorus* | |
| --- | --- | --- | --- | --- | --- | --- | --- | --- | --- | --- | --- | --- | --- |
| Codon | Amino Acid | Count | RSCU | Count | RSCU | Count | RSCU | Count | RSCU | Count | RSCU | Count | RSCU |
| GCA | Alanine (A) | 300 | 1.15 | 303 | 1.15 | 304 | 1.16 | 303 | 1.15 | 303 | 1.16 | 306 | 1.16 |
| GCC | Alanine (A) | 187 | 0.72 | 191 | 0.72 | 189 | 0.72 | 190 | 0.72 | 188 | 0.72 | 186 | 0.71 |
| GCG | Alanine (A) | 118 | 0.45 | 118 | 0.45 | 118 | 0.45 | 118 | 0.45 | 116 | 0.44 | 117 | 0.44 |
| **GCU** | **Alanine (A)** | **440** | **1.68** | **442** | **1.68** | **440** | **1.67** | **441** | **1.68** | **442** | **1.69** | **445** | **1.69** |
| UGC | Cysteine (C) | 95 | 0.81 | 90 | 0.78 | 92 | 0.79 | 91 | 0.78 | 94 | 0.80 | 94 | 0.80 |
| UGU | Cysteine (C) | 140 | 1.19 | 141 | 1.22 | 142 | 1.21 | 142 | 1.22 | 141 | 1.20 | 142 | 1.20 |
| GAC | Aspartic Acid (D) | 146 | 0.51 | 146 | 0.51 | 147 | 0.51 | 145 | 0.50 | 146 | 0.51 | 145 | 0.51 |
| **GAU** | **Aspartic Acid (D)** | **432** | **1.49** | **428** | **1.49** | **429** | **1.49** | **430** | **1.50** | **429** | **1.49** | **429** | **1.49** |
| **GAA** | **Glutamic Acid (E)** | **776** | **1.20** | **773** | **1.20** | **775** | **1.20** | **776** | **1.20** | 775 | 1.19 | **775** | **1.20** |
| GAG | Glutamic Acid (E) | 519 | 0.80 | 520 | 0.80 | 520 | 0.80 | 521 | 0.80 | 523 | 0.81 | 518 | 0.80 |
| UUC | Phenylalanine (F) | 288 | 0.68 | 284 | 0.67 | 284 | 0.67 | 286 | 0.68 | 283 | 0.67 | 286 | 0.68 |
| **UUU** | **Phenylalanine (F)** | **562** | **1.32** | **560** | **1.33** | **559** | **1.33** | **561** | **1.32** | **563** | **1.33** | **558** | **1.32** |
| **GGA** | **Glycine (G)** | **643** | **1.68** | **644** | **1.67** | **644** | **1.68** | **641** | **1.67** | **641** | **1.67** | **643** | **1.67** |
| GGC | Glycine (G) | 363 | 0.95 | 366 | 0.95 | 365 | 0.95 | 366 | 0.95 | 365 | 0.95 | 364 | 0.95 |
| GGG | Glycine (G) | 199 | 0.52 | 198 | 0.51 | 197 | 0.51 | 200 | 0.52 | 200 | 0.52 | 201 | 0.52 |
| GGU | Glycine (G) | 330 | 0.86 | 330 | 0.86 | 330 | 0.86 | 330 | 0.86 | 331 | 0.86 | 332 | 0.86 |
| CAC | Histidine (H) | 299 | 0.64 | 302 | 0.65 | 303 | 0.65 | 302 | 0.65 | 301 | 0.64 | 299 | 0.64 |
| **CAU** | **Histidine (H)** | **635** | **1.36** | **629** | **1.35** | **627** | **1.35** | **630** | **1.35** | **637** | **1.36** | **637** | **1.36** |
| AUA | Isoleucine (I) | 447 | 0.95 | 445 | 0.94 | 444 | 0.94 | 445 | 0.94 | 449 | 0.95 | 449 | 0.95 |
| AUC | Isoleucine (I) | 439 | 0.93 | 436 | 0.92 | 436 | 0.93 | 437 | 0.93 | 439 | 0.93 | 437 | 0.93 |
| AUU | Isoleucine (I) | 523 | 1.11 | 534 | 1.13 | 530 | 1.13 | 531 | 1.13 | 530 | 1.12 | 531 | 1.12 |
| AAA | Lysine (K) | 791 | 1.08 | 777 | 1.08 | 783 | 1.08 | 779 | 1.08 | 783 | 1.08 | 783 | 1.07 |
| AAG | Lysine (K) | 670 | 0.92 | 666 | 0.92 | 668 | 0.92 | 666 | 0.92 | 669 | 0.92 | 675 | 0.93 |
| CUA | Leucine (L) | 307 | 0.92 | 304 | 0.91 | 309 | 0.92 | 304 | 0.91 | 306 | 0.92 | 306 | 0.92 |
| CUC | Leucine (L) | 260 | 0.78 | 258 | 0.77 | 259 | 0.77 | 259 | 0.78 | 258 | 0.77 | 256 | 0.77 |
| CUG | Leucine (L) | 140 | 0.42 | 129 | 0.39 | 129 | 0.39 | 131 | 0.39 | 133 | 0.40 | 134 | 0.40 |
| CUU | Leucine (L) | 208 | 0.62 | 208 | 0.62 | 210 | 0.63 | 209 | 0.63 | 206 | 0.62 | 207 | 0.62 |
| **UUA** | **Leucine (L)** | **497** | **1.49** | **498** | **1.49** | **500** | **1.49** | **496** | **1.49** | **496** | **1.49** | **496** | **1.49** |
| **UUG** | **Leucine (L)** | **596** | **1.78** | **605** | **1.81** | **601** | **1.80** | **601** | **1.80** | **599** | **1.80** | **601** | **1.80** |
| AUG | Methionine (M) | 472 | 1.00 | 469 | 1.00 | 472 | 1.00 | 471 | 1.00 | 474 | 1.00 | 470 | 1.00 |
| AAC | Asparagine (N) | 382 | 1.12 | 386 | 1.11 | 388 | 1.12 | 386 | 1.11 | 386 | 1.11 | 384 | 1.11 |
| AAU | Asparagine (N) | 300 | 0.88 | 309 | 0.89 | 306 | 0.88 | 308 | 0.89 | 308 | 0.89 | 308 | 0.89 |
| **CCA** | **Proline (P)** | **692** | **2.28** | **696** | **2.30** | **694** | **2.30** | **693** | **2.29** | **697** | **2.29** | **696** | **2.30** |
| CCC | Proline (P) | 235 | 0.78 | 233 | 0.77 | 231 | 0.76 | 233 | 0.77 | 230 | 0.76 | 230 | 0.76 |
| CCG | Proline (P) | 162 | 0.53 | 155 | 0.51 | 156 | 0.52 | 156 | 0.52 | 161 | 0.53 | 160 | 0.53 |
| CCU | Proline (P) | 123 | 0.41 | 126 | 0.42 | 127 | 0.42 | 126 | 0.42 | 129 | 0.42 | 124 | 0.41 |
| CAA | Glutamine (Q) | 350 | 0.80 | 354 | 0.81 | 353 | 0.81 | 353 | 0.81 | 355 | 0.81 | 356 | 0.81 |
| **CAG** | **Glutamine (Q)** | **522** | **1.20** | 522 | 1.19 | 523 | 1.19 | 523 | 1.19 | 520 | 1.19 | 524 | 1.19 |
| AGA | Arginine (R) | 189 | 0.96 | 189 | 0.97 | 189 | 0.97 | 188 | 0.96 | 190 | 0.97 | 191 | 0.97 |
| **AGG** | **Arginine (R)** | **363** | **1.85** | **363** | **1.85** | **359** | **1.84** | **361** | **1.85** | **364** | **1.85** | **366** | **1.86** |
| CGA | Arginine (R) | 154 | 0.79 | 156 | 0.80 | 155 | 0.79 | 157 | 0.80 | 156 | 0.79 | 152 | 0.77 |
| **CGC** | **Arginine (R)** | **282** | **1.44** | **279** | **1.42** | **279** | **1.43** | **279** | **1.43** | **279** | **1.42** | **278** | **1.41** |
| CGG | Arginine (R) | 79 | 0.40 | 77 | 0.39 | 77 | 0.39 | 76 | 0.39 | 79 | 0.40 | 77 | 0.39 |
| CGU | Arginine (R) | 110 | 0.56 | 111 | 0.57 | 111 | 0.57 | 111 | 0.57 | 112 | 0.57 | 115 | 0.59 |
| AGC | Serine (S) | 259 | 1.10 | 260 | 1.10 | 259 | 1.10 | 261 | 1.11 | 259 | 1.10 | 260 | 1.10 |
| AGU | Serine (S) | 118 | 0.50 | 121 | 0.51 | 124 | 0.53 | 121 | 0.51 | 120 | 0.51 | 121 | 0.51 |
| **UCA** | **Serine (S)** | **326** | **1.38** | **315** | **1.34** | **316** | **1.34** | **316** | **1.34** | **316** | **1.34** | **321** | **1.36** |
| **UCC** | **Serine (S)** | **315** | **1.34** | **315** | **1.34** | **315** | **1.34** | **315** | **1.33** | **317** | **1.35** | **319** | **1.35** |
| UCG | Serine (S) | 237 | 1.01 | 242 | 1.03 | 241 | 1.02 | 240 | 1.02 | 239 | 1.01 | 236 | 1.00 |
| UCU | Serine (S) | 158 | 0.67 | 162 | 0.69 | 160 | 0.68 | 163 | 0.69 | 162 | 0.69 | 161 | 0.68 |
| **ACA** | **Threonine (T)** | **437** | **1.63** | **436** | **1.64** | **435** | **1.63** | **435** | **1.63** | **434** | **1.63** | **432** | **1.62** |
| ACC | Threonine (T) | 302 | 1.13 | 304 | 1.14 | 302 | 1.13 | 307 | 1.15 | 299 | 1.13 | 301 | 1.13 |
| ACG | Threonine (T) | 205 | 0.76 | 202 | 0.76 | 203 | 0.76 | 203 | 0.76 | 202 | 0.76 | 202 | 0.76 |
| ACU | Threonine (T) | 129 | 0.48 | 124 | 0.47 | 125 | 0.47 | 123 | 0.46 | 127 | 0.48 | 134 | 0.50 |
| **GUA** | **Valine (V)** | **421** | **1.50** | **420** | **1.51** | **423** | **1.51** | **422** | **1.51** | **420** | **1.51** | **414** | **1.49** |
| **GUC** | **Valine (V)** | **414** | **1.47** | **411** | **1.47** | **412** | **1.47** | **410** | **1.47** | **407** | **1.46** | **407** | **1.47** |
| GUG | Valine (V) | 129 | 0.46 | 130 | 0.47 | 129 | 0.46 | 131 | 0.47 | 130 | 0.47 | 132 | 0.48 |
| GUU | Valine (V) | 159 | 0.57 | 155 | 0.56 | 156 | 0.56 | 155 | 0.55 | 157 | 0.56 | 157 | 0.57 |
| UGG | Tryptophan (W) | 422 | 1.00 | 430 | 1.00 | 430 | 1.00 | 429 | 1.00 | 431 | 1.00 | 435 | 1.00 |
| **UAC** | **Tyrosine (Y)** | **353** | **1.45** | **351** | **1.45** | **350** | **1.46** | **351** | **1.46** | **349** | **1.45** | **348** | **1.45** |
| UAU | Tyrosine (Y) | 133 | 0.55 | 132 | 0.55 | 130 | 0.54 | 130 | 0.54 | 131 | 0.55 | 132 | 0.55 |
| **Total Codons:** | | **20,282** |  | **20,260** |  | **20,264** |  | **20,264** |  | **20,286** |  | **20,295** |  |
